# Interplay between Solid Tumors and Tumor Microenvironment

**DOI:** 10.3389/fimmu.2022.882718

**Published:** 2022-05-30

**Authors:** Seung-Jin Kim, Dipendra Khadka, Jae Ho Seo

**Affiliations:** ^1^ Department of Biochemistry, College of Natural Sciences, and Kangwon Institute of Inclusive Technology, Kangwon National University, Chuncheon, South Korea; ^2^ Global/Gangwon Innovative Biologics-Regional Leading Research Center (GIB-RLRC), Kangwon National University, Chuncheon, South Korea; ^3^ NADIANBIO Ltd., Wonkwang University, Business Incubation Center R201-1, Iksan, South Korea; ^4^ Department of Biochemistry, Wonkwang University School of Medicine, Iksan, South Korea; ^5^ Sarcopenia Total Solution Center, Wonkwang University School of Medicine, Iksan, South Korea

**Keywords:** tumor microenvironment, stromal cell, metastasis, tumor heterogeneity, extracellular matrix

## Abstract

Over the past few decades, basic studies aimed at curing patients with cancer have been constantly evolving. A myriad of mechanistic studies on physiological changes and related factors in tumor growth and metastasis have been reported. Recently, several studies have been considerate to how tumors adapt to unfavorable environments, such as glucose deprivation, oxidative stress, hypoxic conditions, and immune responses. Tumors attempt to adapt to unfavorable environments with genetic or non-genetic changes, the alteration of metabolic signals, or the reconfiguration of their environment through migration to other organs. One of the distinct features in solid tumors is heterogeneity because their environments vary due to the characteristics of colony growth. For this reason, researchers are paying attention to the communication between growing tumors and neighboring environments, including stromal cells, immune cells, fibroblasts, and secreted molecules, such as proteins and RNAs. During cancer survival and progression, tumor cells undergo phenotype and molecular changes collectively referred to as cellular plasticity, which result from microenvironment signals, genetics and epigenetic alterations thereby contributing to tumor heterogeneity and therapy response. In this review, we herein discuss the adaptation process of tumors to adverse environments *via* communication with neighboring cells for overcoming unfavorable growth conditions. Understanding the physiology of these tumors and their communication with the tumor environment can help to develop promising tumor treatment strategies.

## Introduction

**Figure 1:**
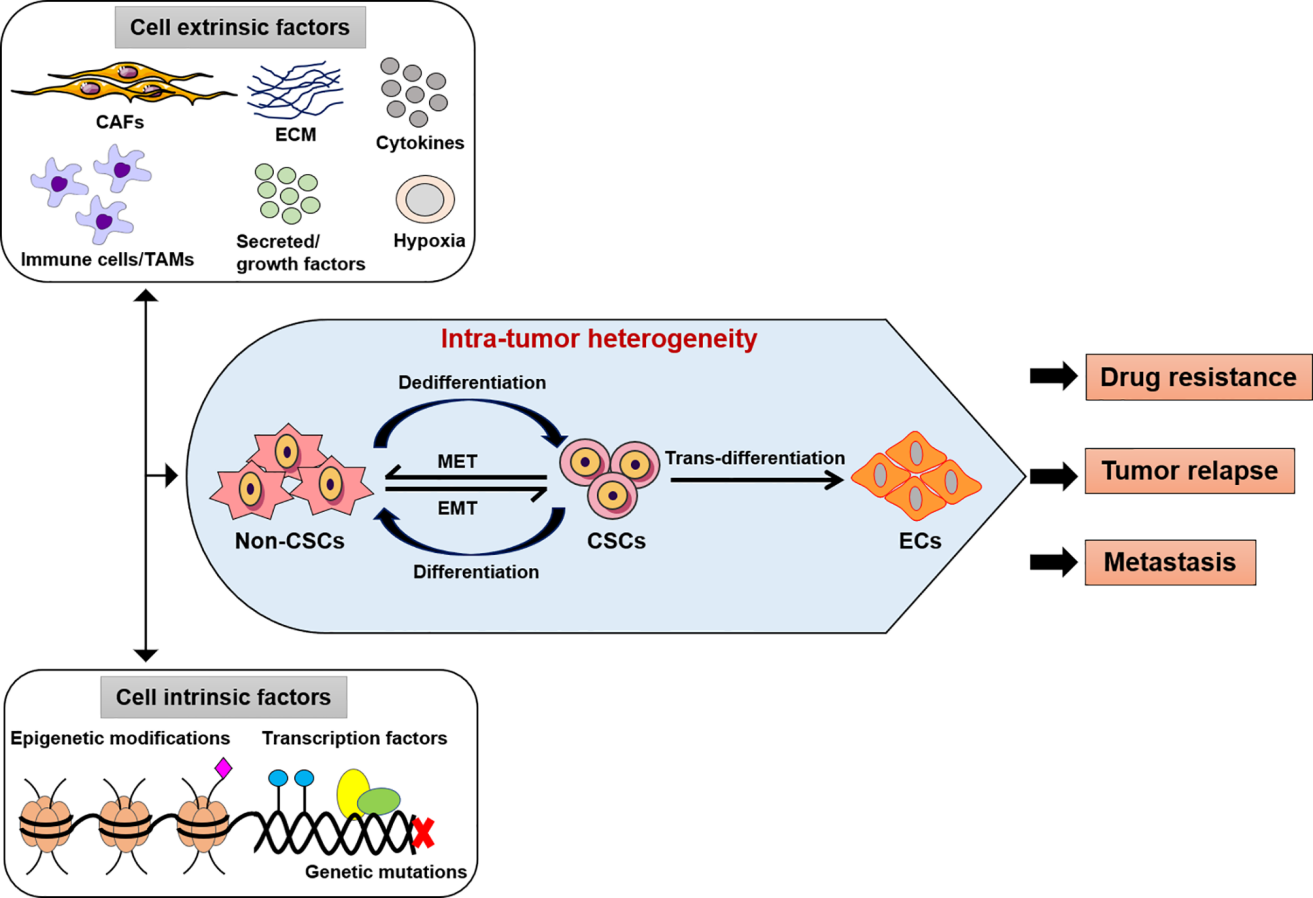
Intra-tumor heterogeneity by extrinsic or intrinsic factors. The modifications of cancer cells such as genetic, epigenetic alterations, and microenvironment perturbations. CSCs show an induced EMT system, which mostly exhibit an intermediate condition. This activity depends on both genetic mutations, epigenetic alterations, and transcriptional modification of cancer cells and signals provided by TME (CAFs or TAMs, immune cells, ECM, cytokines, secreted or growth factors). Thus, the intra-tumor heterogeneity might be play a potential role in the development of effective therapeutic approaches as drug resistance, tumor relapse and metastasis. CSCs, cancer stem-like cells; EMT, epithelial-to-mesenchymal transition; MET, mesenchymal-to-epithelial transition; ECs, endothelial-like cells, CAFs, cancer-associated fibroblasts; TAMs, tumor-associated macrophages; ECM, extracellular matrix.

Understanding of the physiology of solid tumors has changed significantly over the past 30 years. Cancer research has typically focused on the growth and inhibition of primary tumors, but recently more research has focused on the growth and malignancy of tumors through their genetic and non-genetic modification (1, 2). Primary tumors are exposed to various stressful environments, such as oxidative stress, hypoxia, and acidosis, with rapid growth, thereby accelerating their heterogeneity (3, 4). This not only changes the metabolism or genetic modification of the tumor, but also changes the neighboring tumor microenvironment (TME). Conversely, stimulation of the TME promotes changes in tumor development and aggressiveness (**Figure 1**). For this reason, it is necessary to understand communication between tumors and the TME, which includes blood vessels, immune cells, fibroblasts, stromal cells, the extracellular matrix (ECM), and secreted molecules that exist around primary tumors. In this mini-review, we briefly summarize how the interplay between tumors and the TME impacts tumor cell physiology and adaptation for overcoming unfavorable environments.

## Extracellular Matrix

The ECM is a complex ecosystem of various components, such as fibrous proteins (collagen and elastin) or glycoproteins (fibronectin 1, laminins, and tenascin), proteoglycans (chondroitin sulfate and heparan sulfate), and polysaccharides, which includes several growth factors and creates rigid interactions with cancer cells in the TME (5). In the TME, the ECM functions as a framework for the tumor cells and plays an active role in tumor progression, particularly as a vital mediator of invasive processes (6). The ECM performs a tumor-suppressing role in healthy tissues, but it performs a tumor-promoting role in solid tumors. However, numerous effective components in tumor-stimulating roles in the ECM are produced in the TME (7, 8), and they affect cancer cells during interconnection with integrins (9). According to Glasner et al. (10), INF-γ released from intratumoral natural killer (NK) cells alter primary tumor structure by induction of fibronectin 1 in the tumors resulting in restriction of metastases formation. Regulate cancer metastasis formation through stimulating the tumor structure by regulating fibronectin 1 secretion, which is a key component of the ECM. ECM proteins can be formed by numerous stromal cell types and tumor cells, while cancer-associated fibroblasts (CAFs) are a major source for synthesis, assembly, secretion, and alteration of ECM development (11, 12). Besides the intermolecular covalent cross-linkages of ECM, the biophysical characteristics include its rigidity, topography, molecular density, and tension. Thus, the ECM is extremely versatile causing it to experience cellular remodeling under the effect of tumors or tumor stromal cells (13, 14). As the dynamic crosstalk is facilitated by chemokines and growth factors, metastatic circulating tumor cells are secured to and released from the ECM in addition to metabolic changes of the tumor cells. During enlarged tissue rigidity and desmoplasia, the ECM might act as a barrier for drug delivery or gate for opening the basement membrane to promote metastasis (15, 16). Moreover, the ECM of remote tissues or organs could be somewhat formed into permissive soils by circulating tumor cells, soluble factors, or exosomes from primary tumors to mediate the sowing of metastasizing tumor cells (17).

## Stromal Cells

Stromal cells are connective tissue cells, which are one of the key components of cancer progression and regression involved in the TME. They are engaged by tumor cells, and then involve metastasis initiation through the regulation of tumor cells and themselves (18). Glucose deprivation, reactive oxygen species (ROS), hypoxia, and inflammatory signals create unfavorable environments, leading to epithelial-mesenchymal transition (EMT), tumorigenesis, and tumor metastasis (19, 20). These signals are generally accepted that tumor cells alter their microenvironments through the regulation of stromal cells (18). Stromal cells include mesenchymal stem cells (MSCs), fibroblasts, macrophages, endothelial cells (ECs), lymphocytes, and pericytes in tumors, which contribute toward tumor progression and regression (6). The characteristics of cancer are replicative ability, continued angiogenesis, invasion, and metastasis, which are regulated by the interactions within genetically altered cancer and stromal cells. A previous study showed that stromal cells also undergo metabolic changes in the TME, reforming TME metabolism, and translating nutrients into forms that can be absorbed by tumor cells (21).

In the stromal environment, CAFs are the foremost stromal factor of various solid tumors and are also the best-known phenotypic transformers (22). CAFs are a vastly heterogeneous stromal cell population that participates in drug resistance, proliferation, and metastasis in tumor cells *via* the secretion of cytokines and matrix metalloproteinases (MMPs) (18, 22, 23). They promote angiogenesis, ECM remodeling, wound healing, and cancer progression through the regulation of immune systems in immune cells (24). Several key markers are used to identify CAFs, such as fibroblast-specific protein 1, α-smooth muscle actin (α-SMA), platelet-derived growth factor (PDGF) receptor α, and fibroblast activation protein α (FAP-α). Although they include a heterogeneous cell population, the degree of diversity has hardly been studied (25). Therefore, fibroblasts are separated into quiescent fibroblasts and myofibroblasts/CAFs on the basis of distinct expression. In particular, quiescent fibroblasts are less carcinogenic and mostly found in non-malignant tissues, and myofibroblasts or CAFs encourage tumors and trigger tumor relapse along with tumor resistance and are intensely enhanced in metastatic or malignant tumors. Both fibroblast types secrete an exceptional range of elastins and collagens that maintain the ECM, resulting in desmoplasia (26, 27). However, quiescent fibroblasts secrete low levels of collagens (particularly *Col13a1* and *Col14a1*) and high levels of elastins. In addition, myofibroblasts/CAFs are completely derived from tumor tissues and primarily enhanced in collagens and low levels of elastins. CAFs promote angiogenesis, tumorigenesis, and metastasis by secreting pro-inflammatory cytokines and growth factors and enhancing TME remodeling *via* the secretion of ECM components, MMPs, and other molecules (22, 25).

For immune action, CAFs inhibit the activity of recruited lymphocytes and cytotoxic T lymphocytes that form the inflammatory signals to advance tumor progression, and CAFs can rebuild into a pro-metastatic TME from the post-metastatic TME (27). In the context of the TME, the subtypes of CAFs have shown distinct mechanisms of activation, i.e., the stimulation of transforming growth factor (TGF)-β1 or IL-11 and the treatment of IL-1β or IL-6 that activate the upregulation of inflammatory CAF-associated marker genes (28). Furthermore, the differentiation of CAF-related specific markers can result in α-SMA, also called ACTA2, FAP, S100A4, desmin, collagen, and circulating pro-inflammatory cytokines, such as IL-1β, IL-6, IL-8, TGF-β, and CXCL12 (29). CAFs can directly secrete vascular endothelial growth factor (VEGF) in addition to the other growth factors that regulate angiogenesis by suppressing the angiogenesis-blocking role of TSP1 (22). CAFs are additionally typified based on different cellular sources, such as vascular CAFs that originate from perivascular areas, cycling CAFs, matrix CAFs, and developmental CAFs, which are the product of native fibroblasts found in the TME of the genetically engineered MMTV-PyMT breast cancer mouse model (30). According to Brown et al. (31), In a human PDAC model, CAFs are also derived to be immunomodulatory presenting MHCII genes that regulate antigen-specific ligation with CD4^+^ T helper cells by expressing CD74 (32). Despite this, CAFs deviate in metastatic tumors from early-stage tumors, including high metabolic synthesis and released transcriptional profiling. Correspondingly, CAFs release ECM factors that facilitate collagen crosslinking and regulate the survival signals of tumor cells, which immunomodulate the TME avoidance tumor surveillance (22).

MSCs are derived from the umbilical cord, bone marrow, adipose tissue, etc., and form a fibro-vascular network in fibroblasts and vascular pericytes *via* the formation of tumor barrier differentiation. Emerging evidence has strongly suggested that MSCs can be activated by exosomes and participate in the communication of the transfer of proteins in the tumor cells as well as in the stromal cells (24).

Tumor-associated macrophages (TAMs) are the key cells in several types of solid tumors, which can promote tumor progression by generating pro-inflammatory mediators, include cytokines or chemokines, growth factors that alter the tumor-supportive TME and encourage tumor cell proliferation, multidrug resistance and plasticity (33, 34). For instance, NF-ĸB-mediated factors (TNF-α, IL-1β, IL-6, CCL2, CXCL8 and CXCL10) can protect against apoptosis, and pro-angiogenic growth factors (such as VEGF or PDGF, TGF-β and FGF) that adapt tissue architecture and support tumor cell migration, invasion and metastasis (34, 35). In addition, TAMs destabilize local immune surveillance as they can directly decrease T cell and natural killer cell (NK) activities by releasing soluble factors or by expressing cell surface proteins that exhibit the immunosuppressive functions [e.g. arginase 1 (ARG1), indoleamine 2,3-dioxygenase (IDO), programmed death ligand 1 (PD-L1) and TGF-β] or they can indirectly suppress the activities of T cell through the engagement of other immune suppressive cells i.e. regulatory T cells (36, 37). In general, TAMs are a major component of TME that play mutually a significant role as tumor promoters and immune suppressors because they could promote tumor initiation, and act as the fundamental drivers of the immunosuppressive TME, which control the recruitment and function of multiple immune cells.

**Figure 2:**
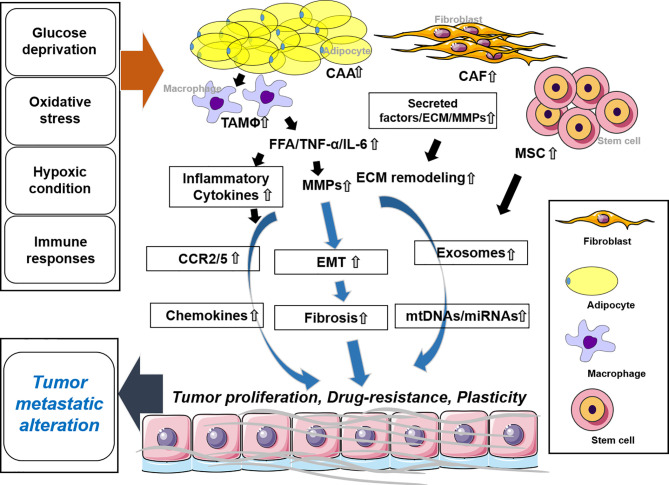
Stromal cells and the tumor microenvironment. CAA regulates EMT by secreting tumor necrosis factor (TNF)-α, IL-6, and FFA along with MMPs. Inflammatory cytokines are secreted by TAM and trigger chemokines. In CAF release, secreted factors and MMPs promote ECM remodeling. MSCs secrete exosomes along with mtDNAs and microRNAs (miRNAs). These molecules synergistically or individually promote tumor proliferation, drug resistance, and plasticity and affect tumor metastatic alteration. CAA, cancer-associated adipocytes; CAF, cancer-associated fibroblast; MSC, mesenchymal stem cell; TAM, tumor-associated macrophage.

Adipocytes are the most abundant cells to compose adipose tissue and they play key roles in energy storage and homeostasis in the body. Cancer-associated adipocytes are key players in cancer progression and migration (38). They highly express matrix remodeling- and EMT-related factors, produce free fatty acids (FFAs) through lipolysis and insulin-like growth factor binding protein 2 (IGFBP-2), and participate in the development of the TME and metastasis (38, 39) (**Figure 2**).

## Immune Cells

In the TME, all immune cells aim to protect the whole body but can ultimately turn into a tumor-supporting cell population (40). Immune cells are remarkably complex and include several different lineages that make them tough to study and target. Depending upon the stage of cancer, both lymphoid and myeloid lineage cells play roles in pro- or anti-tumoral activity. For example, macrophages encourage the activation of T cells to clear tumor cells at early stages but inhibit T cells from even identifying the tumor cells as a tumor growth (41). However, immune cells lead each other to control the mechanisms related to tissue homeostasis and change the survival rate (42). Cellular secretions of molecules from immune cells also influence the activity within the TME. The secretion of cellular molecules, such as CCL5 and XCL1, from NK cells targets antigen-presenting dendritic cells (DCs). Moreover, IFNγ secretion stimulates macrophage polarization and Th1 cell hyperactivation that eventually activates the immune microenvironment against cancer cells (28, 43). In response, cancer cells secrete molecules, i.e., pro-inflammatory cytokines, such as IL-8 and CXCL-1, 2, and 8, that target neutrophils. Despite this, neutrophils generate neutrophil extracellular traps, which protect the cancer cells from NK and cytotoxic CD8^+^ T cells and reduce the influence of immunotherapies (44, 45). Understanding the indispensable role of each immune cell should facilitate the control of immunosuppressive responses and improvement of immunostimulatory functions in secondary tumor proliferation. Considering heterogeneity of immune cells, scRNA-Seq is an advanced technique, which is able to examine the immune cells that show distinct phenotypes *in vivo* models (46).

Monocytes and macrophages are the major phenotypic markers of the aggressive TME (26). In humans, monocytes are subdivided into three largest clusters; namely, classical (CD14^++^, CD16^−^), intermediate (CD14^+^ CD16^+^), and non-classical (CD14^+^ CD16^++^). In tumor cells, recent studies have reported new monocytic markers, such as CD68, CSF1-R, CSF2-R, CD11C, CD1C, CD141, and HLA-DR surface markers (47, 48). TIE2 is a subset of monocytes expressing the angiopoietin receptor that play an important role in tumor angiogenesis, and its expression is highly increased in response to hypoxia (49). Monocytes are absorbed to the TME by chemo-attractants (CCL2 or CCL4), which further differentiate into TAMs. Macrophages are conservatively divided into two main clusters: classical macrophages (CD14+ S100A8/9^+^ M1-like) with antitumor functions and alternate macrophages [CD16^+^(FCGR3A) M2-like] with pro-tumorigenic phenotypes (50). M2 macrophages exhibit the phenotypes of aggressive tumor growth, immune evasion, angiogenesis, and cancer stemness. Furthermore, they assist tumor initiation and the mutagenic microenvironment by releasing circulating pro-inflammatory cytokines (IL6, TNF-α, and IFN-γ), growth factors (VEGF and EGF), ROS, and proteases (51).

The T cell population is usually organized by the cell surface markers (CD3^+^CD4^+^CD8^+^CD25^+^). The complication of tumor-infiltrating T cells indicates a powerful impact of tumors on the T cell transcriptome (52). Conventionally, T cells are categorized into naive, effector, and memory T cells. In lung TME study, single-cell sequencing separated the clusters of T cells into regulatory (FOXP3^+^), CD4^+^ (CD4^+^), CD8^+^ (CD8^+^, naive, effector, memory, or exhausted), NK (FGFBP2^+^), and lesser γδ T cells (26). Naive T cells can be separated into effector T cells following infiltration and further stimulated into cytotoxic memory T cells (53). Mostly, primary tumors are augmented with subtypes of effector T cells that are differentiated by the high expression of chemokine receptors or cytotoxic gene markers (CD28, CD40L, CD137, ICOS, and OX40) and exhibit decreased T cell expression. The expression of co-inhibitory receptors (PD-1, CTLA-4, CD160, LAG3, TIM-3, and TIGIT) leads to progressive T cell dysfunctions with tumor progression from primary to metastatic sites (54). The cells expressing co-inhibitory receptors are immunosuppressive and originate from several sources induced by the TME, including by migration from circulatory systems, effector T cell translation, and separation caused by the inhibition of Antigen Presenting Cells (55).

B cells are adaptive immune cells that infiltrate the TME through CXCL13 secretions from tumor cells (56). In solid tumor tissues, B cells are comparatively plentiful compared to non-tumor tissues (51), and what’s more a relatively rare number of B cells compared to T cells in the TME (28). B cells can be separated into five groups, i.e., plasma B cells expressing IgG (MZB1 and CD138); follicular B cells expressing CD20, CXCR4, and HLA-DRs; mucosa-associated lymphoid tissue-derived plasma B cells expressing IgA (CD38^+^); germinal center B cells; and granzyme B-secreting B cells (26). Although migrating through the germinal center, follicular B cells individually contain mature or naive B cells (CD27^−^, CD72, and IGHM) that result in memory B cells (CD27^+^ and IGHG1) (51). Compared to B cells in the non-tumorigenic environment, B cells residing in the TME are characterized by less protein secretion and the reduction of mTOR or Myc pathways (26). B cells encourage antitumor immunity by motivating complement activation, stimulating cytotoxic immune reactions, antibody-dependent cellular cytotoxicity, phagocytosis, and T cell activation, and releasing granzyme B or TRAIL factors (57). In addition, B cells have immunosuppressive subsets of pro-tumorigenic regulatory B cells (CD1d^+^CD5^+^CD19^+^ and CD5^+^CD19^+^) and CD5^+^ B cells (58) that modulate the production of immunomodulatory cytokines (IL10 and TGFβ), which may enhance metastatic ability by transition of CD4+ T cells into T-reg cells (59).

With the help of a unique set of receptors, NK cells belong to an innate lymphoid cell group and have a cytotoxic or cytokine-producing ability and can recognize tumor cells. However, NK cells are different from the immune cell population as they have diverse cell surface markers (CD3^−^CD16^+^ or CD3^−^CD56^+^). Thus, NK cells are mainly subdivided into distinct subsets depending upon the expression of CD16 and CD56 markers with their different phenotypic properties (60). Tumor-specific NK cells in lung carcinoma reveal the upregulation of CD69 and NKp44 markers and downregulated NKp30, NKp80, DNAM-1, CD16, and ILT2 expression against the peripheral blood and NK cells of normal lung (61). Likewise, DCs have many specific subtypes (DC1, DC2, and CD3) present in the TME that play a significant role in adaptive immune responses, antigen presentation, and phagocytosis. For the other immune cells, the distinguishing markers of DC subsets are HLA DR^+^ lineage^−^ cells, including CD11C^+^ conventional DCs, which are also differentiated into either CD141^+^ or CD1C^+^ cells, and CD123^+^ plasmacytoid DCs (47). DCs are classified based on their presence in lymph nodes or tumor cells. The clusters of tumor cDC1 express Cd103 as a dermal DC marker, while the lymph node population expresses the CD8a marker specific to dendritic populations of the lymph nodes (29).

## Endothelial Cells

Depending on the metabolic needs or requirements of growing tumors, ECs are in a coefficient mode of activation or reactivation and quiescence. The phenotypes of ECs are mostly subdivided into tip and stalk cells that show the different genotypes. Consistent with the tumor requirements, these individual cells adopt distinct phenotypes and functions (62). From the rest of the tumor cells, the first parameter to differentiate ECs is a division through CD45^−^, as a pan-hematopoietic marker that combines with CD31, CD144 (VE-Cadherin), and vWF (von Willebrand Factor). However, CD31 is a transmembrane glycoprotein that develops intercellular intersections. Similarly, CD144 is an endothelial adhesion molecule and vWF is a glycoprotein that mediates platelet adhesion in the endothelium (63). These are the preliminary markers to disconnect the EC population. In contrast, other EC markers in different cancer types comprise tip genes (CLDN5, DLL4, EDNRB, ESM1, KCNE3, NID2, and RAMP3), capillary markers (CA4 and CD36), arterial markers (FBLN5 and GJA5), and ACKR1 gene expression by high endothelial venules, non-myeloid specific marker AIF1, lymphatic markers (PROX1 and PDPN), and pericyte marker RGS5 (64). According to Lambrechts et al. (26), tumor ECs in distinct clusters based on the marker genes are lymphatic ECs (PDPN^+^ and PROX1^+^), tumor-derived blood ECs (FLT1^+^, IGFBP3^+^, and SPRY1^+^), and malignant or non-malignant ECs. In the TME, the dysregulation of epigenetic and transcriptional factors triggers the production of these angiogenic candidates and their subtypes from healthy blood ECs. The subtypes of tumor ECs directly damage the vascular integrity and structure of leaking blood vessels and migration of immune cells, thereby contributing to the growing tumor’s complexity (65).

A previous study investigated the development of the heterogeneity of ECs by determining functionally validated endothelial phenotypes through patients with cancer as well as *in vivo* and *in vitro* models. Compared to aggressive tumors, non-malignant lung tissues have a relatively high profusion of alveolar type II, postcapillary, scavenging capillary, and lymphatic ECs. Even though the phenotypes of the tumor ECs were primarily immature ECs or human-specific lymphatic tumor ECs and tip cells, in tumor or non-tumor tissues, alveolar type II, activated postcapillary vein, and arterial phenotypes are common (66). Goveia et al. also classified the top-ranked marker genes and their specified significant roles in tumor progression as well as in regulating immune surveillance, matrix remodeling, EC migration, and angiogenesis by modulating growth factors and chemical stimuli that activate the angiogenic cascade within the TME, involving fibroblast growth factor (FGF), VEGF, PDGF, TGF-β, TNF, insulin-like growth factor, and MMP (67). In tumor ECs, blood vessels discharge the interconnecting tight junctions, which are complex with high interstitial pressure and are irregularly shaped. However, tumor ECs produce pro-angiogenic growth factors (FGF, VEGF, and PDGF) that exhibit chromosomal abnormalities, which function against cancer therapies (68). In addition, tumor EC-originating cadherin 2 activates VEGF-associated angiogenesis by controlling MAPK/ERK and MAPK/JNK signaling pathways (69).

## Secreted Molecules

Secreted molecules are major factors in the TME function and ECM remodeling, such as cytokines, proteases, integrins, and miRNAs (70). Cytokines are types of proteins that mediate the interaction between cells in the TME, including TNF, interleukins, chemokines, and growth factors, and regulate tumor progression and stromal cells. Moreover, the roles of cytokines in inflammation, apoptosis, tumorigenesis, proliferation, and migration depend on the maintenance of their anti-targets (71). In the TME, extracellular proteolysis acts as a key role that facilitates the proteolysis of the ECM and MMPs among other proteinases, having the nearest connection with tumor progression (72, 73). According to Kessenbrock et al. (73), the degradation of the ECM is mediated by MMPs that promote tumor invasion and metastasis. Additionally, MMPs stimulate tumor growth and angiogenesis as well as regulate apoptosis, whereas the functions of certain MMPs include tumor suppression. Therefore, MMPs are also a set of proteins with inconsistent roles in the TME (74).

Among secreted factors, integrins are essential membrane proteins and cell surface receptors that have an important role in the signaling and transfer of cellular information among cells or between cells and the ECM. In addition, integrins are central to the control of cell-matrix adhesions and play a critical role in the adhesion of circulating tumor cells to original sites leading formation of secondary tumors in TME. However, irregular cell-cell adhesions are a sign of tumors being triggered by disturbed integrins. The expression of metastasis-assisting integrins in the TME is induced, whereas those suppressing proliferation, migration, and survival are inhibited (75). Therefore, integrin expression is usually dysregulated in many solid tumors and play key roles in signaling as well as promotion of tumor cell invasion and migration. Recently, it has emerged that integrins are expressed not only in cells but also in exosomes, which are fundamental units of extracellular vesicles secreted from cells. Numerous studies are concerned with exosome originating integrins as the exploration on exosomes are increasing, in addition integrins are notified to influence the interior actions of tumors, as nucleus alteration. Most of research efforts have focused on supporting incorporation of exosomes by target cells and facilitating exosome-mediated transfer of the membrane proteins and associated kinases to target cells in premetastatic niches. Moreover, integrins have demonstrated the ability to encouraging stem cell-like properties in tumor cells as well as drug resistance (76, 77). miRNAs are endogenous and small non-coding RNAs that negatively regulate specific target mRNAs or post-transcriptionally activate by disordering transcription or translation (78). miRNAs are involved in various pathways and functions in the regulation of distinct constituents of the TME (79). In addition to miRNAs, long non-coding RNAs (lncRNAs) are also effective components that are secreted in the TME. Among lncRNAs, some serve in the interaction between the TME and stromal cells as the transforming fibroblasts that are tumor-promoting (80).

## Conclusion

Despite attempts to discover new anticancer drugs, multidrug resistance and the risk of recurrence remain. In particular, the TME in late-stage tumors is very complex and diverse, thus, it is essential to study the interplay between tumors and the TME for new drug discovery and validation. It is expected that endeavors to understand how tumor cells are reprogramed by communication with adjacent cells and molecules will support the development of new strategies to treat cancers.

## Author Contributions

S-JK designed and wrote the manuscript. DK wrote and reviewed the manuscript. JS supervised the whole project, wrote and reviewed the manuscript. All authors substantially contributed to the article and approved the submitted version.

## Funding

This work was supported by the National Research Foundation of Korea (NRF) grants funded by the Ministry of Science and ICT (2021R1A5A8029876, 2022R1C1C1002819, and 2020R1A5A8019180) and by the Ministry of Education(2021R1I1A3061242).

## Conflict of Interest

Author DK was employed by NADIANBIO Ltd.

The remaining authors declare that the research was conducted in the absence of any commercial or financial relationships that could be construed as a potential conflict of interest.

## Publisher’s Note

All claims expressed in this article are solely those of the authors and do not necessarily represent those of their affiliated organizations, or those of the publisher, the editors and the reviewers. Any product that may be evaluated in this article, or claim that may be made by its manufacturer, is not guaranteed or endorsed by the publisher.
